# Immunogenic cell death as a mechanism of synergy between sunitinib and ^177^Lu-DOTATATE peptide receptor radionuclide therapy in pancreatic neuroendocrine tumors

**DOI:** 10.1038/s41420-026-03344-z

**Published:** 2026-09-19

**Authors:** Markus Essler, Nadine Veit, Aurelia Müller, Milka Marinova, Barbara Kreppel

**Affiliations:** Department of Nuclear Medicine, University Hospital Bonn, Bonn, Germany

**Keywords:** Translational research, Neuroendocrine cancer

## Abstract

Peptide receptor radionuclide therapy (PRRT) with ¹⁷⁷Lu-DOTATATE improves outcomes in neuroendocrine tumors (NETs), yet durable disease control remains limited in progressive pancreatic NET (pNET) and higher-grade disease. Combinations that enhance PRRT efficacy may be advantageous. As sunitinib has radiosensitizing and immunomodulatory effects, we explored the clinical activity and mechanistic basis of combining sunitinib with PRRT. Combination treatment with ¹⁷⁷Lu-DOTATATE (1–3 cycles; 7.4 GBq per cycle) and sunitinib at 37.5 mg/day was well tolerated and effective in a pilot group of heavily pretreated pNET patients with progressive disease. Treatment response was assessed by ⁶⁸Ga-DOTATOC PET/CT and serum tumor markers (CgA, NSE). Mechanistic studies were performed in QGP-1 pNET cells using proliferation and apoptosis assays, cell-cycle analysis, and immunogenic cell death (ICD) markers. Combination therapy was well tolerated and induced marked responses: three patients achieved near-complete responses and two partial responses on post-therapy ⁶⁸Ga-DOTATOC PET/CT. In vitro, ¹⁷⁷Lu-DOTATATE and sunitinib showed bidirectional sensitization: low-dose sunitinib reduced the LD50 of ¹⁷⁷Lu-DOTATATE, and low-activity ¹⁷⁷Lu-DOTATATE reduced the LD50 of sunitinib. ¹⁷⁷Lu-DOTATATE induced dose-dependent PARP cleavage and S/G2–M arrest; however, sunitinib shifted cell death toward late apoptosis/necrosis. The combination produced a more-than-additive increase in calreticulin exposure on the tumor cell surface and enhanced HMGB1 but not interleukin- 1ß- or interleukin-18-release compared with either monotherapy, indicating immunogenic late apoptosis/necrosis, without canonical inflammasome activation. Intermittent sunitinib combined with ¹⁷⁷Lu-DOTATATE PRRT demonstrated encouraging activity in progressive pNET. The mechanism of synergy in vitro was enhanced ICD, suggesting that immune responses contribute to the synergy in vivo.

## Introduction

Neuroendocrine tumors (NETs) comprise a heterogeneous group of malignancies with variable biological behavior, ranging from indolent to highly aggressive phenotypes [1, 2]. Despite advances in systemic therapy, including somatostatin analogs (SSA) [3, 4], targeted agents such as the mTOR inhibitor everolimus, the tyrosine kinase inhibitors sunitinib and cabozantinib [5–7], cytotoxic chemotherapy such as capecitabine, a precursor of 5-fluorouracil [8], and peptide receptor radionuclide therapy (PRRT) with radiolabeled SSA [9], durable disease control in advanced or rapidly progressive disease remains challenging. In particular, pancreatic neuroendocrine tumors (pNETs) and high-grade (G2/G3) NETs frequently exhibit limited response duration to monotherapy, necessitating novel rational combination strategies [10, 11]. For this purpose, therapeutics improving tumor perfusion, upregulating somatostatin receptor (SSTR) expression or blocking DNA repair may be useful. Approved NET therapies, including chemotherapy, multikinase inhibitors, and unlabeled SSA, have been tested in combination with PRRT. A number of retrospective studies report that the combination of PRRT and temozolomide ± capecitabine leads to disease control in 38% to 55% of progressive NETs after failure of PRRT or chemotherapy [12–15]. In particular, combined use of PRRT and chemotherapy seems to be useful in patients with FDG-positive metastases from pNETs [5]. Moreover, combinations of PRRT with molecularly targeted therapeutics such as sunitinib or everolimus were suggested [6] and successfully tested in individual patients. Although no prospective trials studied interactions of PRRT and SSA, a retrospective analysis reports that in a cohort of 168 patients with GEP-NETs who received PRRT, patients who received SSA as maintenance and between PRRT administrations showed significantly longer progression-free survival compared to patients without SSA [15]. These data indicate that SSA as a combination therapy and/or maintenance therapy may play a significant role in tumor control after PRRT [16]. Although a number of combinations of PRRT with other therapeutics were tested, the molecular mechanisms of interaction are unclear. It is widely accepted that irradiation leads to double-strand breaks in the DNA of tumor cells, cell-cycle arrest and, finally, apoptosis [17]. Therefore, the most likely mechanism to enhance the effect of PRRT is sensitization to radiation, for example, by interfering with DNA repair and cell cycle arrest or by enhancement of proapoptotic signaling pathways leading to caspase activation and PARP cleavage. Apoptosis is the most extensively studied cell death mechanism in tumor cells but some isotopes may also cause necrosis, mitotic cell death or senescence in tumor cells [18]. Interestingly, irradiation may also cause immunogenic cell death (ICD). ICD is a form of regulated cell death, in which dying tumor cells emit a set of damage-associated molecular patterns (DAMPs) that convert a silent death into one that functionally activates the immune system [19]. Among these, calreticulin (CRT) externalization is a hallmark of ICD that facilitates phagocytosis. This process licenses dendritic cells for antigen processing and presentation, ultimately promoting cross-priming of tumor-specific CD8⁺ T cells [20, 21]. Another DAMP, high-mobility group box 1 (HMGB1), is released during ICD and acts as a chemotactic agent for natural killer (NK), cytotoxic T, and dendritic cells and thereby propagates antitumor immune reactions [22]. Radiation may also induce pyroptosis by inflammasome activation and subsequent formation of cytokines such as interleukin 18 (IL-18). Radiation in the form of β-emission from ¹⁷⁷Lu has multiple prerequisites for ICD induction: it provokes endoplasmic reticulum stress, generates reactive oxygen species, and causes persistent DNA injury, all of which are upstream triggers of CRT externalization [23, 24]. In the context of PRRT, this sequence may be particularly relevant because NETs often exhibit low intrinsic immunogenicity (“cold tumors”), and any therapy able to convert apoptotic clearance into immunogenic clearance may act as an immunologic amplifier [25, 26]. By making irradiated NET cells more visible to the immune system through calreticulin exposure, ¹⁷⁷Lu-DOTATATE may overcome part of the immunologic inertia that characterizes these tumors. Sunitinib plays an important role in the treatment of NET [27] and was reported to exert immunomodulatory activity in vitro and in vivo [28–30]. Sunitinib is also a radiosensitizer, potentially enhancing the effect of PRRT in NET patients.

## Results

### Combination treatment of progressive pancreatic NET with sunitinib and ^177^Lu-DOTATATE

**Fig. 1:**
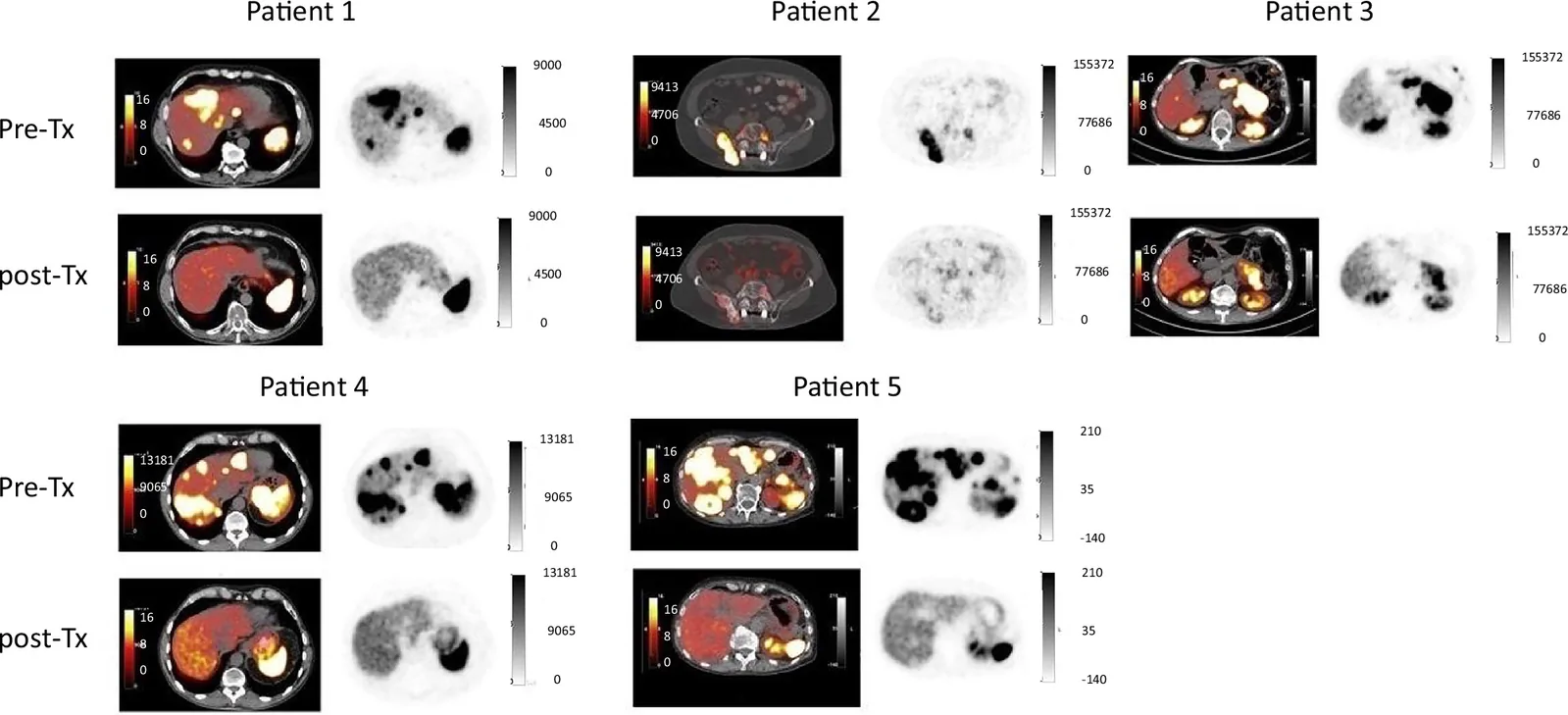
Response to combined sunitinib and ^177^Lu-DOTATATE treatment in patients with progressive pancreatic neuroendocrine tumors. Representative ⁶⁸Ga-DOTATOC PET images obtained before treatment and 8 weeks after the final cycle of PRRT are shown for all five patients in the pilot cohort. Patients received 1–3 cycles of ¹⁷⁷Lu-DOTATATE (7.4 GBq per cycle) at 12-week intervals, with sunitinib administered at a dose of 37.5 mg/day between PRRT cycles. Follow-up imaging demonstrated a marked reduction in the extent and intensity of somatostatin receptor–positive tumor lesions in all patients.

**Fig. 2:**
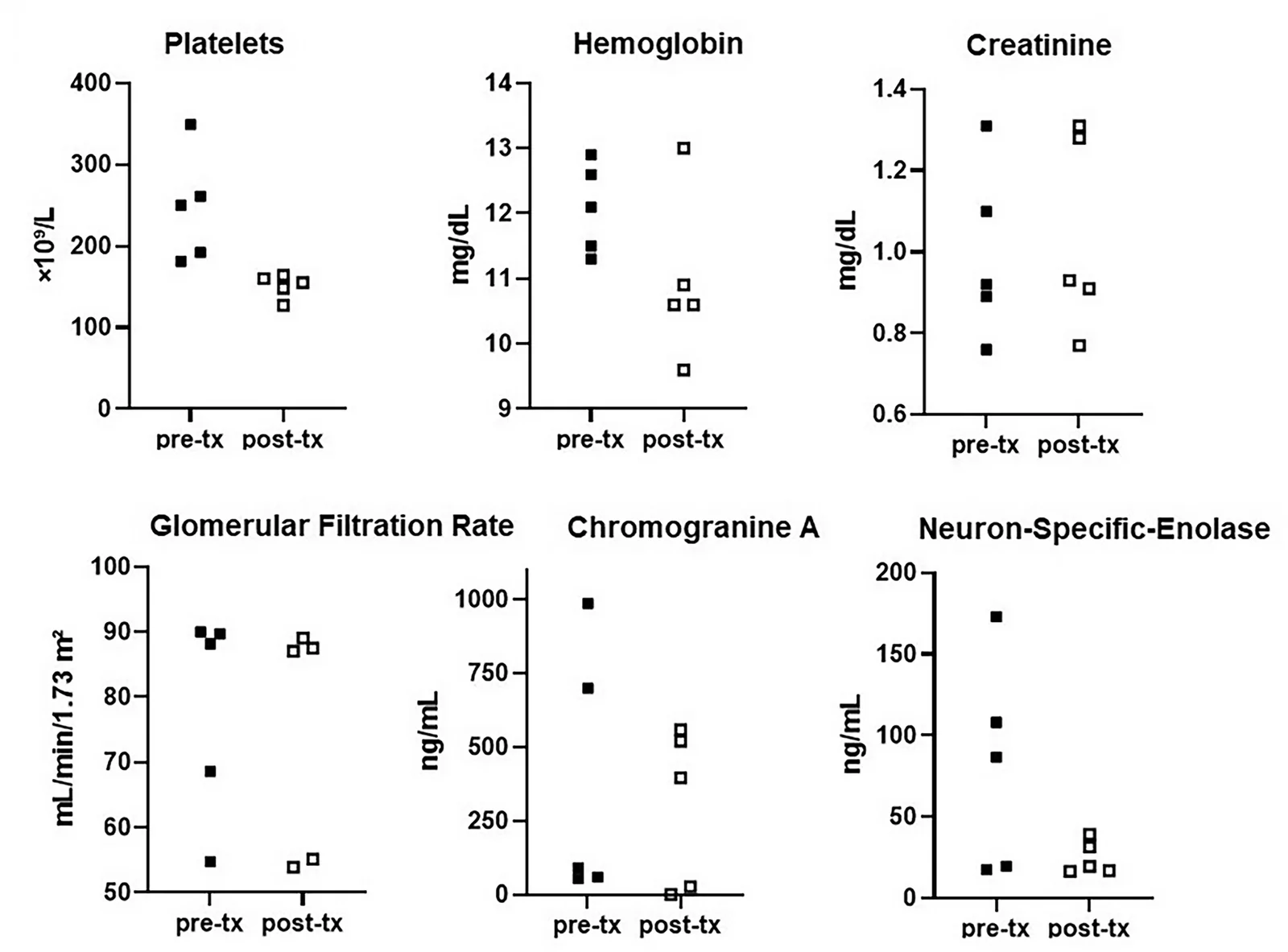
Changes in tumor markers, hematologic parameters, and renal function following combined sunitinib and ^177^Lu-DOTATATE treatment. Paired dot plots show serum chromogranin A (CgA) and neuron-specific enolase (NSE) levels, hemoglobin concentrations, platelet counts, serum creatinine levels, and estimated glomerular filtration rates (eGFR) before and after combination treatment. Black squares indicate pretreatment values and white squares indicate post-treatment values.

We treated five patients with metastatic pancreatic NET G2 or G3, high tumor burden, and disease progression under PRRT, chemotherapy, or everolimus with a combination of PRRT (1–3 × 7.4 GBq ¹⁷⁷Lu-DOTATATE) and sunitinib (37.5 mg/d) in the intervals between ¹⁷⁷Lu-DOTATATE administrations. The decision to treat was made in an interdisciplinary tumor conference of the Center for Integrated Oncology (CIO) of the University Hospital Bonn. Clinical and biographical data of the patients are shown in Table 1. For therapy planning and response assessment, we performed ⁶⁸Ga-DOTATOC PET before therapy and 8 weeks after the final application of PRRT. On follow-up PET, we found a near-complete response in three patients and a partial response in two patients (Fig. 1). This is surprising, as all patients had previously shown progression under PRRT or even chemotherapy. Consistent with response to therapy, levels of the tumor markers neuron-specific enolase (NSE) and CgA were lower after this treatment (Fig. 2). The combination therapy was tolerated well. Side effects are shown in Table 1. The sunitinib dose was reduced to 25 mg/d if necessary. We monitored laboratory parameters before and after therapy. Figure 2 shows that creatinine and glomerular filtration rates (GFR) were not clearly reduced by the treatment. In contrast, platelet counts and hemoglobin values were reduced in some patients after treatment. These observations suggest a synergy between ¹⁷⁷Lu-DOTATATE and sunitinib in the treatment of NETs. To elucidate the underlying mechanisms, we studied the potential synergy in vitro.

**Table 1:** Biographical and clinical data of the patients treated with sunitinib and ¹⁷⁷Lu-DOTATATE.

Patient #	Age	Sex	Previous treatment	Stage	Cycles Sunitinib + PRRT	Side effects	Best Response (PET)
1	52	Female	Biotherapy, 1 × PRRT	cTx, M1 G2 (ki67 15%)	2	none	partial
2	73	Male	Biotherapy, 6 × PRRT	pTx M1 N0 M1 G2 (ki67 10%)	4	dyspnoe	nearly complete
3	69	Male	Biotherapy, Carboplatin, 4 × PRRT	cTx M1 G2 (Ki67 20%)	2	bloody stool, diarrhea, meteorism, spasmodic hickup	nearly complete
4	49	Male	Denusomab, Capecitabin, Temozolomid	pT3 N1 M1 G2 (ki 67 8%)	3	osteonecrosis	partial
5	68	Male	Biotherapy, Denusomab, Everolimus, 4 × PRRT	pTx M1 N0 M1 G2 (10%)	2	none	partial

### Sunitinib and ^177^Lu-DOTATATE show synergistic effects on cell proliferation in vitro

**Fig. 3:**
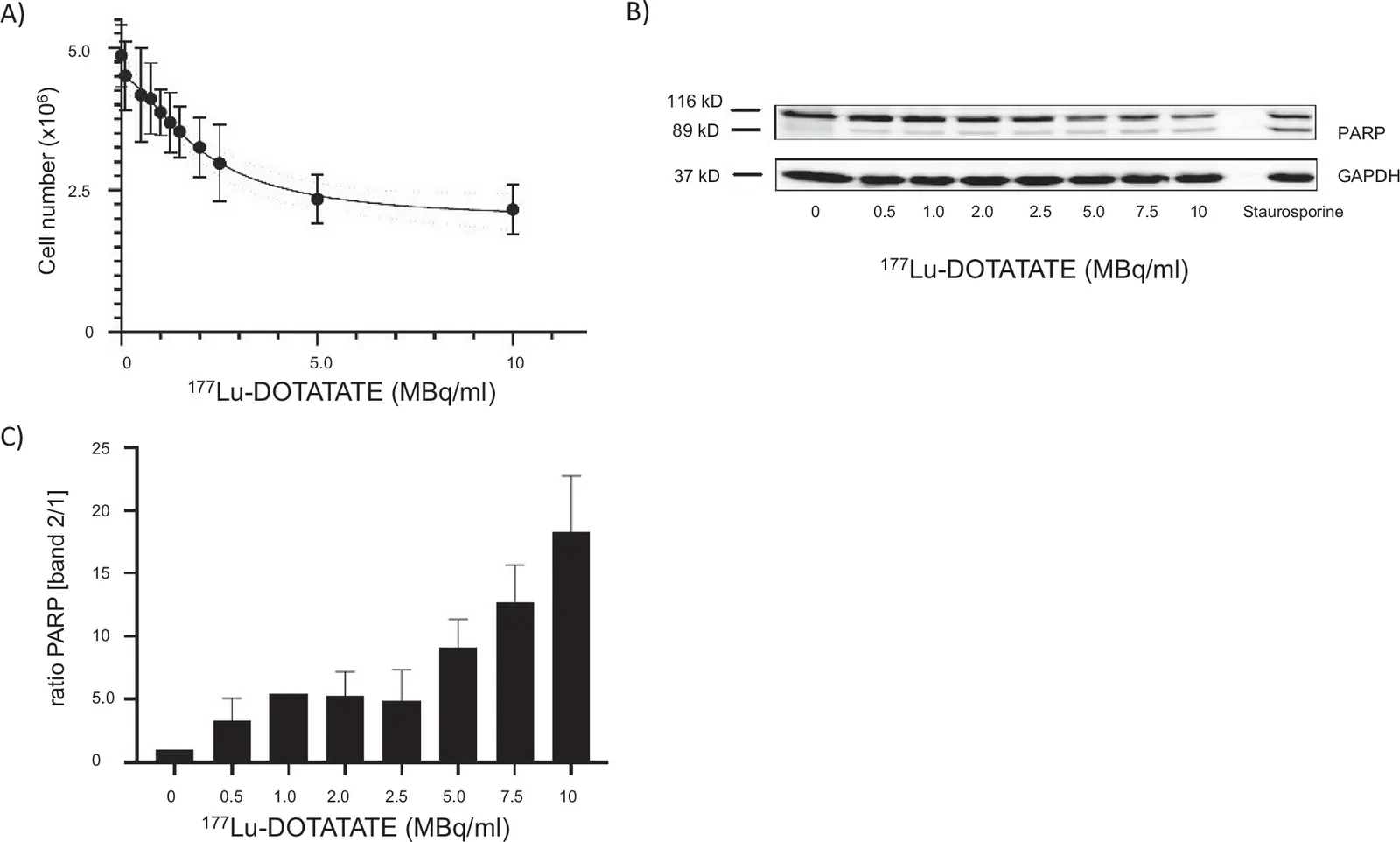
^177^Lu-DOTATATE inhibits proliferation and induces PARP cleavage in QGP-1 cells in an activity-dependent manner. **A** Proliferation of QGP-1 cells following treatment with increasing activities of ¹⁷⁷Lu-DOTATATE. Cell numbers are presented as mean ± standard error of the mean (SEM). **B** Representative Western blot showing full-length PARP (116 kDa) and cleaved PARP (89 kDa) after treatment with increasing activities of ¹⁷⁷Lu-DOTATATE. Staurosporine was used as a positive control for apoptosis induction. **C** Densitometric quantification of the ratio of cleaved to full-length PARP. Data are presented as mean ± standard deviation (SD) from three independent experiments. The difference between untreated control cells and cells treated with 10 MBq/mL ¹⁷⁷Lu-DOTATATE was assessed using Student’s *t* test.

We studied the effect of ¹⁷⁷Lu-DOTATATE and sunitinib on the proliferation of QGP-1 pancreatic NET cells. ¹⁷⁷Lu-DOTATATE (1–10 MBq/mL) inhibited cell proliferation in a dose-dependent manner (Fig. 2). We next studied whether ¹⁷⁷Lu-DOTATATE induces poly(ADP-ribose) polymerase (PARP) cleavage as an indicator of apoptosis in QGP-1 cells. Western blots using antibodies to 116-kDa (full-length protein) and 89-kDa (cleaved fragment) of PARP showed that ¹⁷⁷Lu-DOTATATE (0.5–10 MBq/mL) induced cleavage of PARP in a dose-dependent manner (Fig. 3). Densitometric analysis showed that the level of cleaved PARP corresponded with the activity of ¹⁷⁷Lu-DOTATATE applied (Fig. 3). The activity of ¹⁷⁷Lu-DOTATATE reducing proliferation to 50% of controls (LD50) was 1.4 MBq/mL and the LD50 of sunitinib was 7.43 µM (Table 2). Incubation of cells with a low-activity sunitinib concentration (2.5 µM = 0.3 × LD50) reduced the LD50 of ¹⁷⁷Lu-DOTATATE to 0.88 MBq/mL. Reduction of LD50 indicates enhancement of the antiproliferative effect. On the other hand, pretreatment of cells with very low activities of ¹⁷⁷Lu-DOTATATE (0.5 MBq/mL = 0.3 × LD50) reduced the LD50 of sunitinib to 2.47 µM, indicating that ¹⁷⁷Lu-DOTATATE also enhances the antiproliferative effect of sunitinib (Table 2). These data are consistent with our clinical finding that ¹⁷⁷Lu-DOTATATE and sunitinib act synergistically on the proliferation of pancreatic NET cells.

**Table 2:** LD50 values of ¹⁷⁷Lu-DOTATATE, sunitinib or combinations of both therapeutics.

	LD50	LD50	LD50
	**Single Substance**	**+Sunitinib (2.5 µM)**	+**177Lu-DOTATATE (0.5 MBq/ml)**
^**177**^**Lu-DOTATATE**	1.4 ± 0.1 MBq/ml	0.8 ± 0.1 MBq/ml	
**Sunitinib**	7.43 ± 0.456 µM		2.67 ± 0.1 µM

### ^177^Lu-DOTATATE induces apoptosis in a dose-dependent manner

One possible explanation for the synergistic effect of ¹⁷⁷Lu-DOTATATE and sunitinib may be increased levels of apoptosis. Densitometric analysis of the protein bands reacting with anti-PARP antibody indicated that cleavage was dose-dependent, as the ratio of cleaved to intact PARP increased with higher activities of ¹⁷⁷Lu-DOTATATE (Fig. 3).

### Sunitinib enhances ^177^Lu-DOTATATE-induced necrosis

**Fig. 4:**
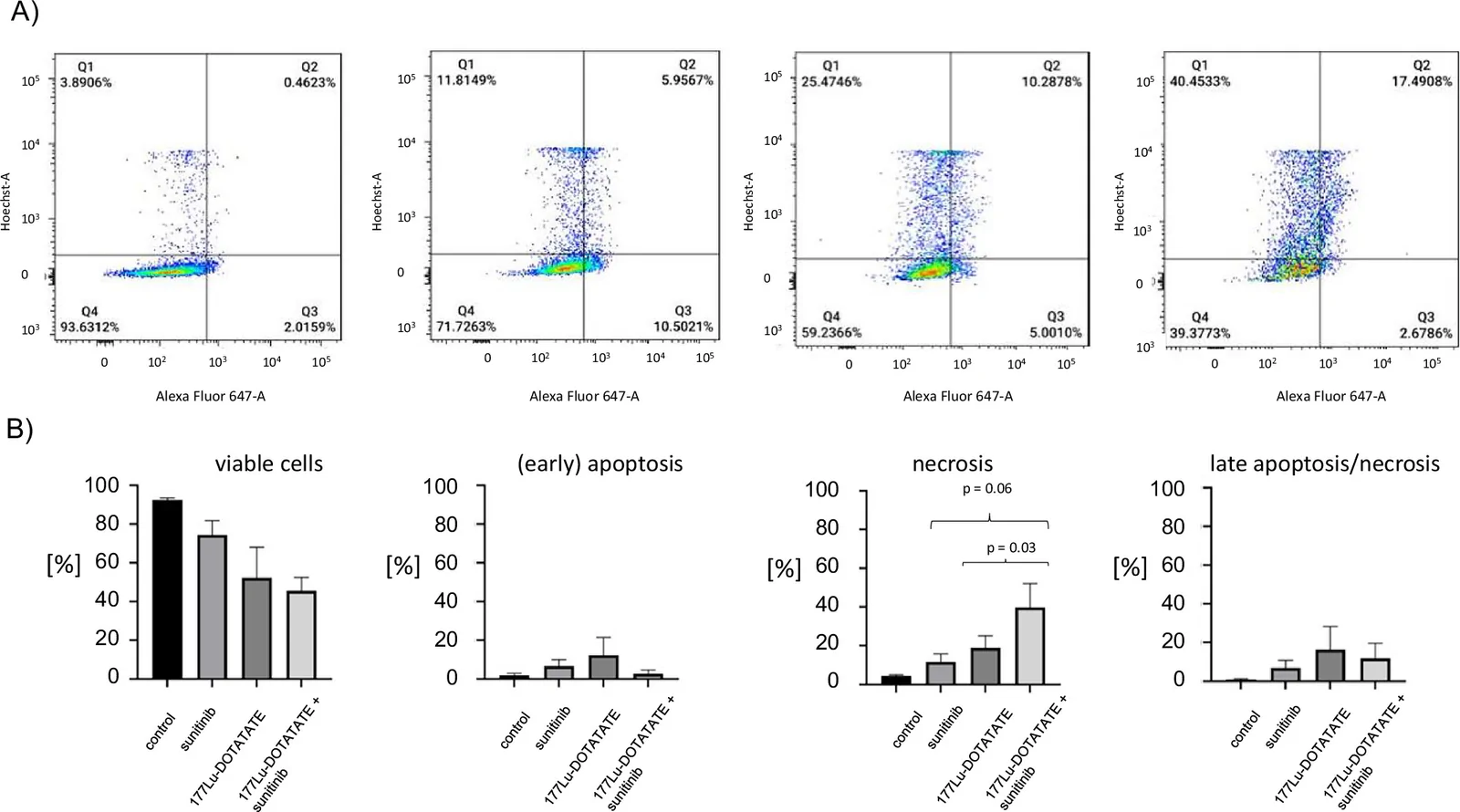
Combined treatment with sunitinib and ^177^Lu-DOTATATE increases necrotic cell death in QGP-1 cells. **A** Representative flow cytometry plots of QGP-1 cells treated as indicated and stained with annexin V and Hoechst 33342. Quadrants were defined as follows: Q1, necrotic cells; Q2, late apoptotic/necrotic cells; Q3, early apoptotic cells; and Q4, viable cells. **B** Quantification of viable, early apoptotic, late apoptotic/necrotic, and necrotic cells following treatment with sunitinib, ¹⁷⁷Lu-DOTATATE, or their combination, compared with untreated controls. Data are presented as the mean percentage ± SD. ANOVA demonstrated a significantly higher proportion of necrotic cells after combination treatment than after sunitinib alone (*P* = 0.006) or ¹⁷⁷Lu-DOTATATE alone (*P* = 0.03).

For further elucidation of cell death mechanisms induced by ¹⁷⁷Lu-DOTATATE and sunitinib, we stained cells with FITC-annexin V and Hoechst 33342 and assessed apoptosis, late apoptosis, and necrosis by flow cytometry. Viable cells were negative for both dyes. Apoptotic cells are positive for FITC-annexin V. Necrotic cells are positive for Hoechst 33342. Late apoptotic cells are positive for both dyes. Treatment of cells with ¹⁷⁷Lu-DOTATATE or sunitinib reduced the levels of viable cells but increased the levels of apoptotic and necrotic cells. Combination treatment further reduced the percentage of viable cells but increased the percentage of necrotic cells characterized by Hoechst 33342 staining. This difference was statistically significant as tested by ANOVA analysis (^177^Lu-DOTATATE vs. combination: *p* = 0.03; Sunitinib vs. combination: *p* = 0.006). The percentage of aopototic cells was not increased in a statistically significant manner by the combination compared to each single treatment (Fig. 4). Hoechst 33342 staining indicates disintegration of the plasma membrane. These data suggest that the synergy of ¹⁷⁷Lu-DOTATATE and sunitinib may not be due to enhanced levels of apoptosis, but rather to increased levels of necrosis or to faster progression of initially apoptotic cells into necrosis.

### ^177^Lu-DOTATATE induces cell-cycle arrest in the S and G2/M phases

**Fig. 5:**
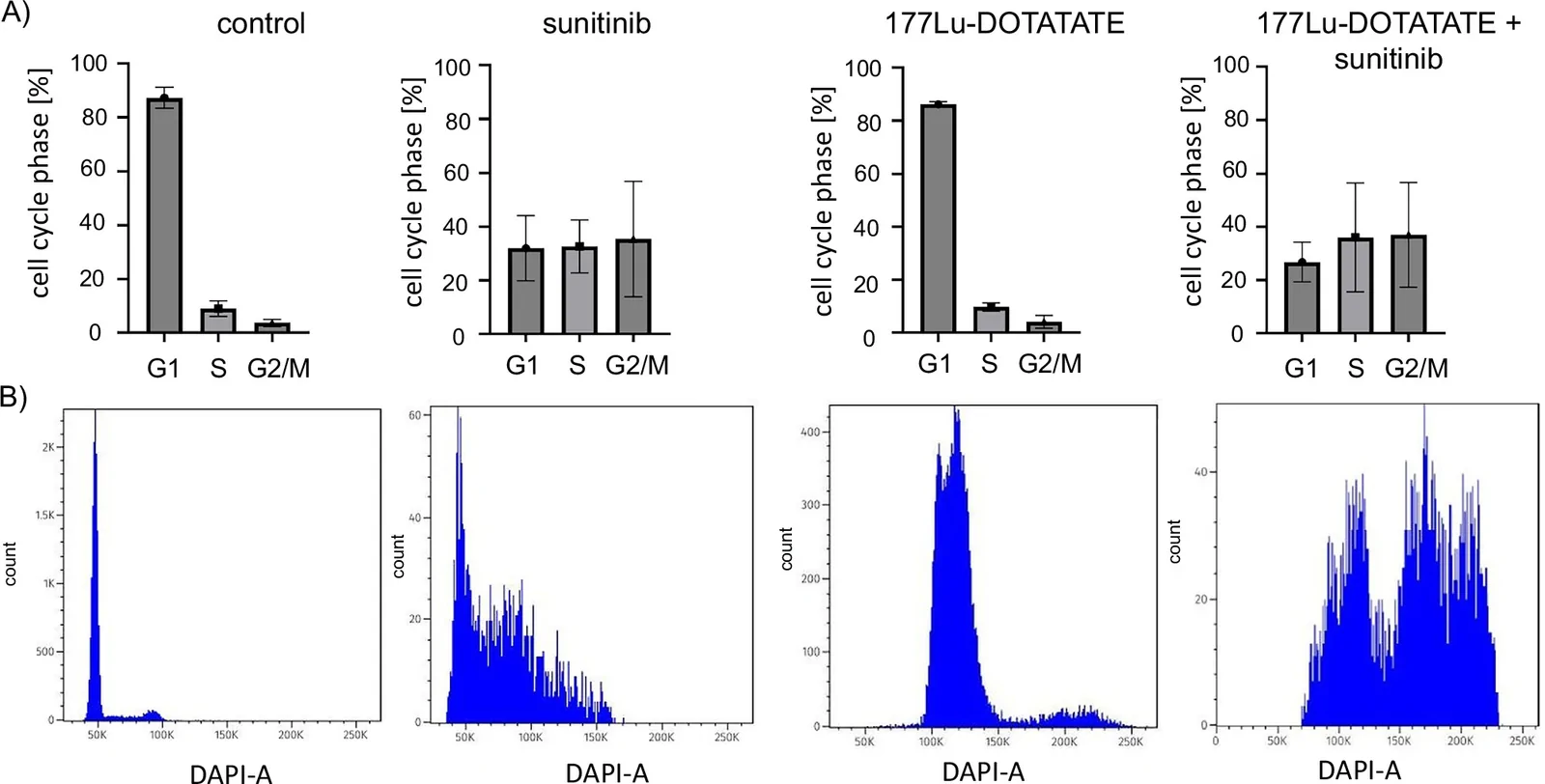
^177^Lu-DOTATATE induces accumulation of QGP-1 cells in the S and G2/M phases of the cell cycle. QGP-1 cells were treated for 5 days with ¹⁷⁷Lu-DOTATATE (10 MBq/mL), sunitinib (7.5 µM), or their combination. Cells were stained with propidium iodide and Hoechst 33342, and cell-cycle distribution was analyzed by flow cytometry. **A** Quantification of the proportions of cells in the G0/G1, S, and G2/M phases. **B** Representative DNA-content histograms for the indicated treatment groups. Data are presented as mean ± SD from three independent experiments. The differences in S- and G2/M-phase between control and ^177^Lu-DOTATATE-treated cells was statistically significant in a student’s *T*-test: S-phase: *P* = 0.065; G2/M-phase: *P* = 0.043).

As described above, our data indicate that sunitinib promotes the effect of ¹⁷⁷Lu-DOTATATE on cell proliferation by increased levels of necrosis. It is possible that this is not the only mechanism of interaction of the two therapeutics. For example, combination treatment may also inhibit proliferation by a cell-cycle blockade. Therefore, we studied the cell cycle distribution of QGP-1 cells after treatment with ¹⁷⁷Lu-DOTATATE, sunitinib, or a combination of both. In controls, approximately 85% of cells were in the G0/G1 phase, 10% in the S phase, and 5% in the G2/M phase. Sunitinib (5 µM) did not change the distribution in a statistically significant manner. In contrast, after treatment with ¹⁷⁷Lu-DOTATATE (5 MBq/mL) only about 30% of cells were in the G0/G1 phase, 30% in the S phase, and 40% in the G2/M phase of the cell cycle. After treatment with ¹⁷⁷Lu-DOTATATE and sunitinib, this distribution of cells within the cell cycle was not significantly changed (Fig. 5). These data indicate that ¹⁷⁷Lu-DOTATATE induces a cell-cycle arrest in the S and G2/M phases that was not further enhanced by sunitinib.

**Fig. 6:**
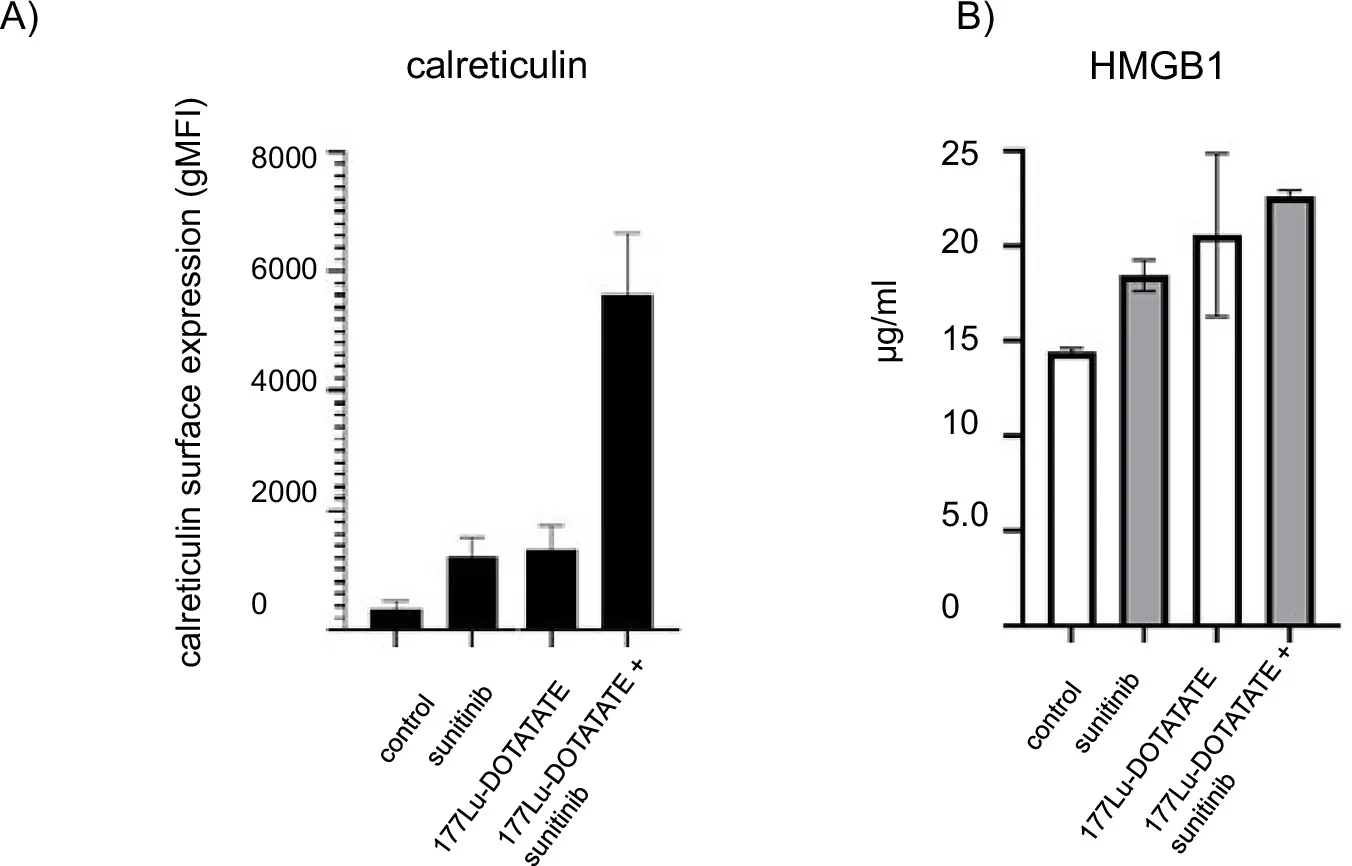
Combined treatment with sunitinib and ^177^Lu-DOTATATE enhances markers of immunogenic cell death in QGP-1 cells. Cells were treated for 5 days with ¹⁷⁷Lu-DOTATATE (10 MBq/mL), sunitinib (7.5 µM), or their combination, as indicated. **A** Cell-surface calreticulin expression was quantified by flow cytometry. Geometric mean fluorescence intensity = gMFI. **B** HMGB1 concentrations in culture supernatants were measured by ELISA and are expressed in µg/mL. Data are presented as mean ± SD from three independent experiments. Combination treatment significantly increased both calreticulin surface expression and HMGB1 release compared with untreated controls (Student’s *t* test, *P* < 0.001 for HMGB1 and *p* = 0.03 for Calreticulin).

Sunitinib enhances ¹⁷⁷Lu-DOTATATE-mediated ICD. Another mechanism by which sunitinib may enhance the activity of ¹⁷⁷Lu-DOTATATE in pancreatic NETs is activation of the immune system by ICD. Two hallmarks of ICD are translocation of calreticulin to the cell surface and release of HMGB1 into the extracellular space. We used flow cytometry to determine calreticulin on the surface of QGP-1 cells. In untreated control cells, a low level of calreticulin was present on the cell surface. ¹⁷⁷Lu-DOTATATE and sunitinib both upregulated calreticulin. Remarkably, treatment with ¹⁷⁷Lu-DOTATATE and sunitinib enhanced this effect more strongly, indicating a synergistic effect of both agents. Similarly, only very low concentrations of HMGB1 were found by ELISA in the supernatants of QGP-1 control cells or after treatment with ¹⁷⁷Lu-DOTATATE or sunitinib. In supernatants from cells treated with both agents, HMGB1 was present in much higher concentrations (Fig. 6). In contrast, IL-18 was not present in the supernatants under any of these conditions (data not shown). We used a cytokine ELISA array to assess other cytokines potentially secreted in the supernatants upon treatment with Sunitinib and ^177^Lu-DOTATATE. We did not find enhanced secretion of interleukin-1ß or other cytokines upon combination treatment in in our system. These findings indicate that sunitinib and ¹⁷⁷Lu-DOTATATE synergize to induce cell death by immunogenic late apoptosis/necrosis but not by canonical inflammasome activation.

## Discussion

PRRT with ¹⁷⁷Lu-DOTATATE improves disease control and survival in patients with advanced, somatostatin receptor–positive NETs. Nevertheless, therapeutic options remain limited for patients with progression during or after PRRT and for those with aggressive or higher-grade disease. Identifying agents that increase the antitumor activity of ¹⁷⁷Lu-DOTATATE is therefore of considerable clinical interest. In this exploratory study, five patients with progressive pancreatic NETs received combined treatment with ¹⁷⁷Lu-DOTATATE and sunitinib. Three patients achieved near-complete metabolic responses and two achieved partial responses. Treatment-related adverse effects were manageable, although the sunitinib dose had to be reduced in two patients. No clinically relevant nephrotoxicity was observed. Although these findings require confirmation in larger prospective cohorts, they suggest that the combination of sunitinib and PRRT may represent a promising therapeutic strategy for selected patients with treatment-refractory pNET.

Our in vitro experiments provide evidence for a direct interaction between sunitinib and ¹⁷⁷Lu-DOTATATE. Low-dose sunitinib reduced the activity of ¹⁷⁷Lu-DOTATATE required to inhibit QGP-1 cell proliferation by 50% by approximately 38%. Conversely, low-activity ¹⁷⁷Lu-DOTATATE markedly reduced the concentration of sunitinib required to achieve comparable growth inhibition. These reciprocal effects support a more-than-additive interaction between the two agents.

Treatment with ¹⁷⁷Lu-DOTATATE induced PARP cleavage and increased the proportion of annexin V–positive cells, consistent with the induction of apoptosis. Combination treatment with sunitinib reduced the proportion of cells displaying an early apoptotic phenotype while increasing the fraction of cells with loss of plasma membrane integrity. This pattern may indicate accelerated progression from apoptosis to late apoptosis or secondary necrosis rather than induction of a distinct primary necrotic pathway. Importantly, membrane permeabilization was accompanied by enhanced exposure of calreticulin on the cell surface and increased extracellular release of HMGB1. These findings are compatible with the induction of ICD.

Calreticulin exposure represents an important “eat-me” signal that promotes recognition and phagocytosis of dying tumor cells by antigen-presenting cells. Both ¹⁷⁷Lu-DOTATATE and sunitinib alone increased surface calreticulin, whereas the combination produced a clearly greater-than-additive effect. Similarly, substantial HMGB1 release was predominantly observed after combined treatment. Thus, the enhanced therapeutic activity may not simply result from an increased number of membrane-compromised cells but may also involve qualitative changes in the immunogenicity of tumor-cell death. Nevertheless, our experiments did not directly assess dendritic-cell activation, phagocytosis, antigen presentation, or tumor-specific T-cell responses. The functional relevance of the observed DAMPs, therefore, remains to be established.

Notably, combined treatment did not induce detectable release of either interleukin-1β or interleukin-18. Because maturation and secretion of these cytokines are characteristic downstream consequences of canonical inflammasome activation, their absence argues against activation of a canonical inflammasome pathway in our experimental system. The observed phenotype therefore appears to represent an inflammasome-independent form of ICD characterized by calreticulin exposure and HMGB1 release without measurable IL-1β or IL-18 secretion. This distinction is relevant because ICD is not a uniform process. The spectrum of exposed or released danger signals depends on the tumor-cell type, the mode of cell death, and the stimulus inducing cellular injury. Similar variability has been reported for other tyrosine kinase inhibitors, including cabozantinib, which can induce calreticulin exposure and HMGB1 release in tumor cells, although the magnitude of these responses differs among cell lines.

The antiproliferative interaction between sunitinib and ¹⁷⁷Lu-DOTATATE was not explained by an additional effect on cell-cycle distribution. ¹⁷⁷Lu-DOTATATE caused a pronounced reduction in the G0/G1 fraction and accumulation of cells in the S and G2/M phases, consistent with a radiation-induced DNA-damage response. Sunitinib alone did not significantly alter cell-cycle distribution and did not further enhance the ¹⁷⁷Lu-DOTATATE-induced S- or G2/M-phase accumulation. Enhancement of cell-cycle arrest is therefore unlikely to be the principal mechanism underlying the combined effect.

Our findings differ from those of Delbart et al., who observed no significant alteration of cell-cycle distribution in six SSTR-expressing cancer cell lines 10 days after exposure to ¹⁷⁷Lu-DOTATATE [31]. This discrepancy may reflect differences in tumor-cell lineage, SSTR2 density and functionality, radiopharmaceutical uptake and intracellular retention, absorbed dose, and, importantly, the timing of cell-cycle analysis. In our experiments, the pronounced accumulation of QGP-1 cells in the S and G2/M phases may represent an earlier or more persistent response that was no longer detectable at the late assessment time point used by Delbart et al. These methodological and biological differences should be considered in comparing the cellular effects of ¹⁷⁷Lu-DOTATATE across experimental models [31].

This study has several limitations. The clinical observations in a collective of five patients are retrospective and exploratory. Therefore, robust conclusions regarding efficacy, safety, response duration, progression-free survival, overall survival, and generalizability cannot be drawn. Moreover, the relative contributions of direct cytotoxicity, vascular effects, altered tumor-cell signaling, and immune-mediated mechanisms remain unclear. The in vitro experiments were performed in a single pNET cell line and did not reproduce the complexity of the tumor microenvironment or interactions with immune and stromal cells. Furthermore, calreticulin exposure and HMGB1 release are consistent with ICD but do not by themselves demonstrate a functional antitumor immune response.

Prospective clinical trials are required to determine whether combined treatment with sunitinib and ¹⁷⁷Lu-DOTATATE improves response duration, progression-free survival, or overall survival compared with established treatment strategies. Future translational studies should include longitudinal analyses of immune-related gene expression, proteomic profiles, circulating immune mediators, and tumor-infiltrating immune-cell populations in patient-derived material. Functional experiments assessing dendritic-cell activation, phagocytosis, and tumor-specific T-cell responses will be particularly important to determine whether the observed molecular phenotype translates into effective antitumor immunity.

In conclusion, our findings support the hypothesis that sunitinib sensitizes pNET cells to ¹⁷⁷Lu-DOTATATE-induced damage and promotes an immunogenic secondary necrotic phenotype. Enhanced calreticulin exposure and HMGB1 release may facilitate immune recognition of dying tumor cells. The absence of IL-1β and IL-18 release indicates that this effect occurs without detectable canonical inflammasome activation. Together with the encouraging clinical responses observed in this small pilot cohort, these results provide a rationale for prospective evaluation of sunitinib plus ¹⁷⁷Lu-DOTATATE in patients with advanced, treatment-refractory pNET.

## Methods

### Positron emission tomography (PET) imaging

The mean injected activity was 200 MBq [⁶⁸Ga]Ga-DOTATOC, and the mean time interval from tracer injection to image acquisition was 45 min ([⁶⁸Ga]Ga-DOTATOC). PET/CT examinations were performed on a Vereos digital PET/CT scanner (Philips Healthcare, Eindhoven, Netherlands). Low-dose CT scans were performed for attenuation correction. Emission time per bed position was 2 min. PET data were reconstructed iteratively, including scatter, random, and decay correction (RAMLA 3D, 2 iterations, 0.05 relaxation).

### ^177^Lu-DOTATATE treatment

Treatment was performed as described previously [15]. Patients received a nephroprotective infusion of amino acids (2.5% lysine and 2.5% arginine in 1 L 0.9% NaCl) for over 4 h, starting 30 min before therapy with antiemetic premedication (ondansetron, 8 mg i.v.).

### Cell culture

The NET cell line QGP-1 was obtained from the Japanese Cancer Research Resources Bank (JCRB, JCRB0183, Ibaraki, Osaka, Japan). Cells were cultured in RPMI 1640 medium (Thermo Fisher Scientific, Waltham, MA) containing 10% FBS, 2 mM L-glutamine, and 100 U/mL penicillin-streptomycin.

### Cell proliferation assay

Cells were seeded in 24-well tissue culture plates (Sarstedt, Nümbrecht, Germany) with four replicates per experimental condition. Cells were plated at 7.5 × 10⁴ cells per well in complete growth medium and allowed to attach (day 0). Sunitinib (Selleck Chemicals LLC, Houston, TX) or ¹⁷⁷Lu-DOTATATE was added in various concentrations or doses as indicated to the appropriate wells (day 1). ¹⁷⁷Lu-DOTATATE was removed after 24 h and, after washing twice with PBS (Thermo Fisher Scientific, Waltham, MA), replaced with complete growth medium or, in combination experiments, medium containing the specified concentration of sunitinib (day 2). The medium was replaced midway through the experiment (day 6). On day 12 after seeding, plates were fixed and stained with 0.05% (w/v) crystal violet in 20% (v/v) methanol for 20 min at room temperature. Plates were then gently rinsed twice in a basin with tap water to remove excess stain and inverted to air dry overnight. After drying, 1.0 mL of methanol was added to each well and plates were briefly placed on an orbital shaker when needed to fully solubilize the bound dye. The dye was measured at 540 nm using a plate reader. For each condition, the mean absorbance was calculated from the four replicate wells and the mean blank value from wells containing medium only (processed and stained identically but without cells) was subtracted from each mean before further analysis. For calculation of cell numbers from OD values, individual calibration curves were determined for each experiment using GraphPad Prism (GraphPad Software, LLC, Version 10.0.3, San Diego, CA).

### Flow cytometry

QGP-1 cells were seeded in 6-cm dishes (Sarstedt, Nümbrecht, Germany) at least one day prior to treatment. Cells were treated with 10 MBq/mL ¹⁷⁷Lu-DOTATATE for 24 h. Control cells were left untreated. Following incubation, cells were washed twice with phosphate-buffered saline (PBS), replenished with fresh culture medium and incubated for an additional 4 days, either with or without 7.25 µM sunitinib. After 5 days of total incubation, cells were detached using Accutase (Sarstedt, Nümbrecht, Germany), washed with PBS, and transferred into FACS tubes (Sarstedt, Nümbrecht, Germany). For calreticulin detection, detached cells were incubated with PE-Cy7-conjugated anti-calreticulin antibody (Cell Signaling Technology, Danvers, MA) diluted 1:50 in staining buffer (2 mM EDTA, 0.5% bovine serum albumin, and 0.09% sodium azide in PBS) (Millipore, Darmstadt, Germany). Incubation time was 30 min at room temperature in the dark. Then, cells were centrifuged and washed twice with staining buffer. The final cell pellet was resuspended in 150–250 µL staining buffer for flow cytometry analysis. Fluorescence intensity was measured using BD FACSCanto™ II (Becton, Dickinson (BD), Franklin Lakes, NJ). The data were analyzed using BD FlowJo v10 Software with regard to the geometric mean of the fluorescence intensity (gMFI) of the stained cell populations. For quantification of apoptotic cells, we used Annexin V-Elab Fluor® 647 Reagent (Elabscience Biotechnology, Houston, TX). In brief, harvested cells were resuspended in Annexin V-binding buffer (containing calcium ions). Annexin V-Elab Fluor® 647 was added according to the manufacturer’s instructions and incubated for 15 min at room temperature in the dark. To distinguish apoptotic stages, the membrane-impermeable DNA dye Hoechst 33258 (Thermo Fisher Scientific, Waltham, MA) was added simultaneously. For cell-cycle analysis, cells were fixed with 2% (PFA, methanol-free, Polysciences, Warrington, PA) on ice for 10 min with occasional gentle mixing. Cells were permeabilized with ice-cold 0.1% Triton X-100 (PanReac AppliChem, Darmstadt, Germany) in PBS on ice for 5 min. For DAPI (4′,6-diamidino-2-phenylindole, Sigma-Aldrich, St. Louis, MO) staining samples were resuspended in 250–500 µL DAPI working solution (3.5 µg/mL in PBS) and incubated for 10 min at room temperature. For flow cytometry, we used a BD FACSCanto II (Becton, Dickinson and Company, Franklin Lakes, NJ). DNA histograms were plotted on a linear scale and cell cycle fractions (percentages of cells in G0/G1, S, and G2/M phases) were quantified using BD FlowJo v10 Software upon cell doublet, aggregate, and debris discrimination via pulse processing.

### Western blot

Apoptosis was also determined by Western blot analysis. Cell culture preparations were made as described for flow cytometry. Protein extraction, total protein determination, sample preparation, SDS gel electrophoresis, and Western blotting were performed as previously described. Membranes were blocked in blocking/incubation buffer (5% Amersham ECL blocking agent (Amersham Biosciences, Little Chalfont, Buckinghamshire, UK) in TBS (Carl Roth GmbH, Karlsruhe, Germany) containing 0.1% Tween-20) for 60 min at room temperature. Primary antibodies against PARP and GAPDH were rabbit monoclonal antibodies (mAbs) from Cell Signaling Technology (Danvers, MA) and diluted 1:1000 as indicated by the manufacturer in the aforementioned blocking/incubation buffer. Membranes were incubated overnight at 4°C with antibody, washed three times with incubation buffer and incubated for 60 min with peroxidase-labeled secondary antibody (Peroxidase-AffiniPure Goat Anti-Rabbit IgG (H + L), Jackson ImmunoResearch Laboratories Inc, West Grove, PA), diluted 1:10,000 in incubation buffer. For development of blots, ECL solution (SuperSignal™ West Pico PLUS Western Blotting Substrate, Thermo Fisher Scientific, Waltham, MA) and an Amersham™ Imager 600 were used (Amersham Biosciences, Little Chalfont, Buckinghamshire, UK). ImageJ (release 1.53r, Bethesda, MD, USA) and Microsoft Excel (release 2019, Microsoft Corporation) were used for semiquantitative analysis.

### ELISA assays

Cells were seeded in 24-well format at least the evening before. At approximately 90% confluence, cells were treated with sunitinib and/or ¹⁷⁷Lu-DOTATATE for 24 h with the indicated concentrations. The cell culture supernatant was collected and processed according to the manufacturer’s protocol. The Human HMGB1 (Elabscience, Houston, TX) and IL-18 ELISA kit (Quantikine™ ELISA Human Total IL-18 Immunoassay, R&D Systems, Minneapolis, MN) were used.

### Cytokine Array Kit

For assessment of secretion of cytokines into cell supernatants we used the Proteome Profiler^TM^ Array Human XL Cytokine Array Kit, R&D Systems, Minneapolis, MN. Cells were seeded in 24-well format at least the evening before. At approximately 90% confluence, cells were treated with sunitinib and/or ¹⁷⁷Lu-DOTATATE for 24 h with the indicated concentrations. The cell culture supernatant was collected and processed according to the manufacturer’s protocol.

### Patient characteristics

All patients were treated at the University Hospital Bonn, Comprehensive Cancer Center. Biographical and clinical data are shown in Table 1.

### Clinical chemistry

Chromogranin A (CgA), NSE, GFR, platelet counts and Hemoglobin values from patient blood samples were determined in the department of clinical chemistry in the University Hospital Bonn as part of the clinical treatment monitoring according to ISO 15189 and CLSI international clinical chemistry guidelines.

### Statistics

Statistical analyses were performed using GraphPad Prism version 10.0.3 (GraphPad Software, LLC, San Diego, CA, USA). Differences among experimental groups in ELISA and flow cytometry experiments were assessed using analysis of variance (ANOVA). Densitometric data were analyzed using Student’s *t* test. All statistical tests were two-sided, and *P* values < 0.05 were considered statistically significant.

## Supplementary information

Supplemental File Western Blot Figure 3

## Data Availability

All data generated or analyzed are included in this article. Further inquiries can be directed at the corresponding author.
